# Evaluation of the Fill-it-up-design to use historical control data in randomized clinical trials with two arm parallel group design

**DOI:** 10.1186/s12874-024-02306-2

**Published:** 2024-09-09

**Authors:** Stephanie Wied, Martin Posch, Ralf-Dieter Hilgers

**Affiliations:** 1 Institute of Medical Statistics, RWTH Aachen University, Aachen, Germany; 2 Medical University of Vienna, Centre for Medical Data Science, Institute of Medical Statistic, Vienna, Austria

**Keywords:** Randomized clinical trial, Historical control, External controls, Type I error probability, Power, Sample size, Equivalence

## Abstract

**Purpose:**

In the context of clinical research, there is an increasing need for new study designs that help to incorporate already available data. With the help of historical controls, the existing information can be utilized to support the new study design, but of course, inclusion also carries the risk of bias in the study results.

**Methods:**

To combine historical and randomized controls we investigate the Fill-it-up-design, which in the first step checks the comparability of the historical and randomized controls performing an equivalence pre-test. If equivalence is confirmed, the historical control data will be included in the new RCT. If equivalence cannot be confirmed, the historical controls will not be considered at all and the randomization of the original study will be extended. We are investigating the performance of this study design in terms of type I error rate and power.

**Results:**

We demonstrate how many patients need to be recruited in each of the two steps in the Fill-it-up-design and show that the family wise error rate of the design is kept at 5$\%$. The maximum sample size of the Fill-it-up-design is larger than that of the single-stage design without historical controls and increases as the heterogeneity between the historical controls and the concurrent controls increases.

**Conclusion:**

The two-stage Fill-it-up-design represents a frequentist method for including historical control data for various study designs. As the maximum sample size of the design is larger, a robust prior belief is essential for its use. The design should therefore be seen as a way out in exceptional situations where a hybrid design is considered necessary.

**Supplementary Information:**

The online version contains supplementary material available at 10.1186/s12874-024-02306-2.

## Introduction

The traditional way to demonstrate efficacy of an intervention compared to a control in clinical research is to carry out a randomized controlled trial (RCT) [28]. The control group enables the researcher to differentiate between the effects of the intervention compared to the response under control treatments [14]. Concurrent enrollment, randomization and blinding are tools to ensure comparability of the treatment and the control group and to mitigate bias in the treatment effect estimate. On the other hand control groups, which are observed outside the randomized clinical trial, so called *historical controls*, can systematically differ from the control group of patients within the randomized clinical trial, e.g. due to time trends. These could be caused, for example, by a change in the standard of care, a change in the patient population, or other external changes [2, 24]. However, in the context of rare diseases, it is challenging to carry out a well powered RCT due to small populations. In this situation, the EMA/CHMP guideline [5] on the conduct of clinical trials in small populations states, that in the background of small and very small populations, less conventional approaches may be acceptable if the interpretability of study results can be improved. A guidance document from the FDA [7] also indicates that

$"\ldots$ a hybrid approach of using external control data to add to a concurrent randomized control arm in a clinical trial may sometimes be useful."

Combining data from an RCT with historical controls may favor sample size reduction and allocation of more patients to the experimental group by implementing unbalanced allocation. A central point of discussion is the similarity of the estimated treatment effect estimated with or without historical controls. Consequently, there exists an interest in the development of methods to combine historical controls with randomized controls. A major concern is that the treatment effect estimate may be biased when borrowing historical controls [2]. To control for potential bias, it is required that the historical controls are sufficiently similar to the randomized controls. Different ways to use the historical control data have been suggested with a strongly increased interest in the last decades. Pocock [21] discussed the idea early on and pointed out that the use of historical instead of randomized controls allows one to assign all future patients to the newly supposed superior treatment. However, this comes with the risk of introducing biases. Many different approaches have been proposed, including Bayesian methods like Power Priors, modified Power Priors [3, 11, 13], probability weighted Power Prios [1], commensurate priors [11, 12], as well as meta-analytic predictive approaches [10, 26] like the random-effects model [19]. Pocock’s bias model [21] is also based on a Bayesian approach. The Power Prior [6] approach can also be used to determine the sample size needed in the new trial and to estimate the impact of the historical controls on the power of a clinical trial [4]. In Bayesian power prior methods, the similarity of the controls is taken into account by quantifying a parameter for heterogeneity, thereby performing dynamic borrowing [9, 20]. Furthermore, the available Bayesian methods have been compared concerning bias, precision, power and type I error rate [29].

Approaches based on Frequentist methods are however sparse. Viele et al. [30] introduced different methods to borrow information from historical data, ranging from naive pooling of historical and current controls to complete separate analyses, ignoring the historical controls, as well as intermediate approaches as the test-then-pool approach. The goal of all these methods is to increase the power of the randomized clinical trial, without increasing the sample size [30]. Controversial discussions on the use of historical controls are still ongoing. Kopp-Schneider [16] pointed out that the historical control data may not be viewed as a random sample and thus power and type I error restrictions have to be investigated on the uniform most powerful test.

For rare diseases, where the population size is limited and large RCTs are not feasible, early decision for borrowing is needed. In this light, we consider a stepwise adaptive approach for the inclusion of historical controls. In the first step, a small randomized trial is performed whose sample size is chosen under the assumption that the historical controls can be included in the analysis. If this is not possible, because the historical and current controls differ substantially, the second step is conducted, continuing randomization to increase the sample size to deliver the requested level of evidence. To summarize, we design a clinical trial with a mid-inspection of whether the historical controls are sufficiently similar to the randomized controls. With this paper we propose the *Fill-it-up*-design, evaluate the properties to combine historical data with new randomized data and develop recommendations on the use of this design.

The paper aims to provide clear conditions for the application of the *Fill-it-up*-design while adhering to predefined type I error and type II error probabilities. Therefore the paper is structured as follows. In the section entitled “Statistical model” we describe the *Fill-it-up*-design. In the section entitled “Evaluation of the Fill-it-up-design” sample size calculations of and relations between the individual tests and derivations of the type I and type II error probabilities are presented. In the section entitled “Comparison with Bayesian approach”, we present a comparison of the preceding investigations with the Bayesian MAP approach followed by an illustration of a clinical trial conducted with external data using the *Fill-it-up*-design in the context of an exemplary study on Friedreich’s ataxia in the section entitled “The Fill-it-up-design in practice”. Finally, in the “Recommendations” section we give recommendations regarding the inclusion of historical registry data. A discussion is given in the “Conclusion” section.

## Statistical model

**Fig. 1:**
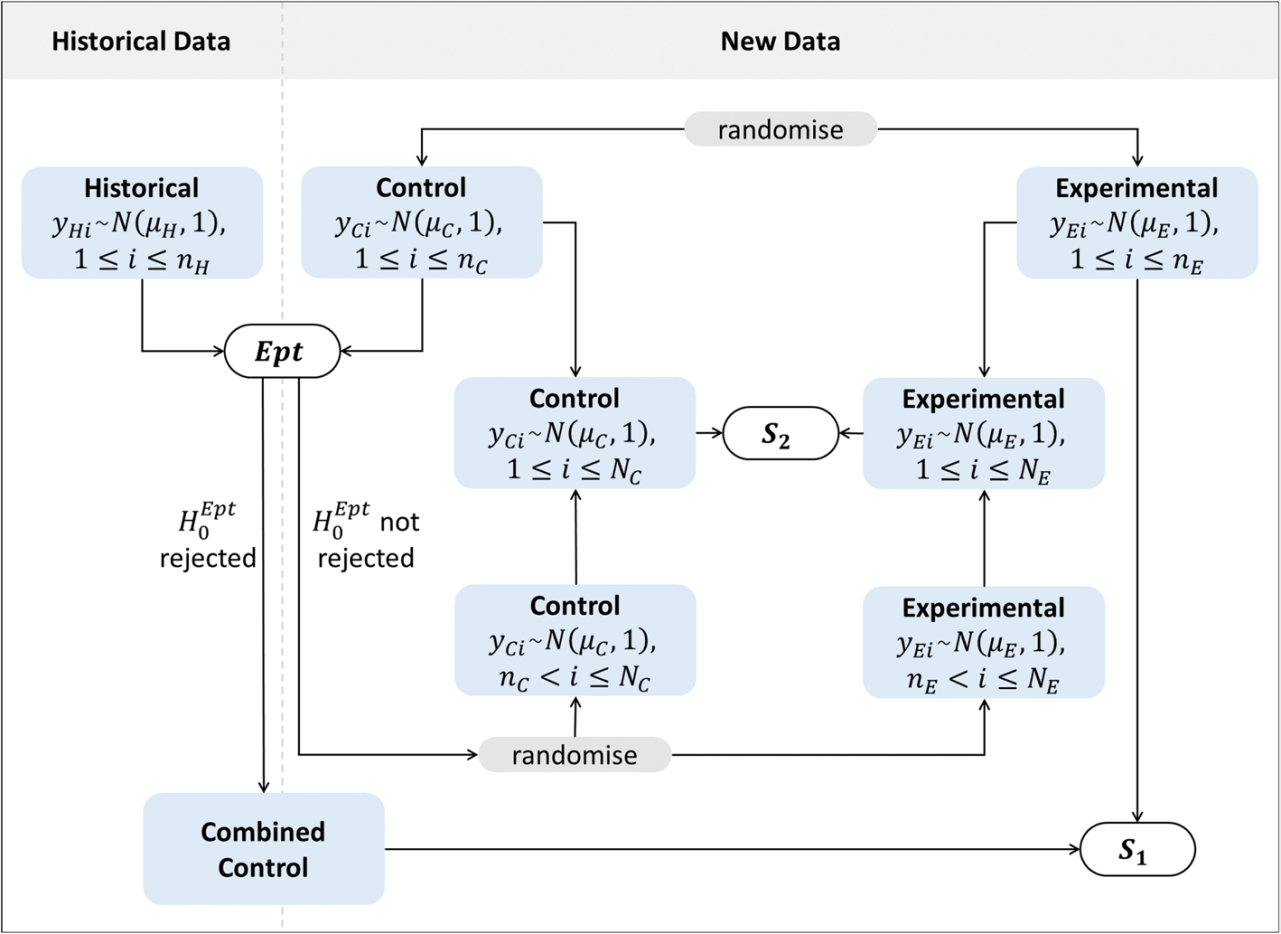
Flow chart of the procedure of the Fill-it-up-design

In the following, we consider a randomized single-center clinical trial with a two-arm parallel group design without adaptation of the randomization procedure. The aim is to investigate whether the experimental treatment *E* is superior to the control treatment *C* concerning a continuous normal endpoint. To determine whether pooling is reasonable, an interim inspection compares the response data of historical and randomized control patients. Figure 1 displays the procedure of the *Fill-it-up*-design.

### Statistical model - notations of the Fill-it-up-design

The responses of the experimental treatment *E* and the control treatment *C*, respectively, in a two arm, parallel group clinical trial are measured with the continuous normally distributed endpoint variable $y_{ji}, 1\le i \le n_j$ with $j \in \{ E,C\}.$ A third group of patients, denoted as the *historical control group* (*H*) is supplemented to the randomized trial, which consists of $n_{H}$ patients from an “external database”. We consider this as a random sample from a control data population. This group provides data of a continuous normally distributed endpoint variable $y_{Hi}, 1\le i \le n_H$, for the response and is supposed to be comparable to the $n_{C}$ control patients. Comparability will be specified below. The expected responses under *E*, *C* and *H* are denoted by $\mu _E$, $\mu _C$ and $\mu _H$ respectively. For simplicity, we assume that the variances of the responses are $\sigma _E^2 =\sigma _C^2= \sigma _H^2 = 1.$ The expected responses are estimated by the corresponding sample means $\bar{y}_E$, $\bar{y}_C$ and $\bar{y}_H$. In the second step of the trial, the randomization process is continued, if comparability of $\mu _C$ and $\mu _H$ could not be established in an equivalence pre-test (*Ept*). Then, further $n_{E}'$ and $n_{C}'$ patients are enrolled in the trial providing estimated responses $\bar{y}'_E$ and $\bar{y}'_C$ of the $N_E=n_{E} + n_{E}'$ and $N_C=n_{C} + n_{C}'$ observations. We assume that the sample means are unbiased estimates, meaning $E(\bar{y}'_E) = E(\bar{y}_E) = \mu _E$ and $E(\bar{y}'_C) = E(\bar{y}_C) = \mu _C$.

### Statistical model - test statistics of the Fill-it-up-design

According to Fig. 1 the two step *Fill-it-up*-design involves three tests. First, an equivalence pre-test to establish the comparability of the randomized control group and the historical control group using $n_{C} + n_{H}$ observations. If the historical control group is found to be comparable, the control data is pooled to test the superiority of the experimental group *E* versus the combined control group based on $n_{E}$ and $n_{C} + n_{H}$ patients. If the historical control group is not found to be comparable with the randomized controls, recruitment of the randomized part of the study is continued with $n_{C}'$ and $n_{E}'$ patients and the test on the superiority of *E* versus *C* is based on $N_E=n_{E} + n_{E}'$ and $N_C=n_{C} + n_{C}'$ patients. Hereby, the power of the pooled superiority test ($S_1$) as well as the superiority test with continued recruitment ($S_2$) is assumed to be $(1-\beta )$ to detect the assumed effect size $\delta$.

#### Equivalence test

After enrollment of the $n_E+n_C$ patients in the randomized part of the first step, a pre-test (*Ept*) to show equivalence with the equivalence margin $\Delta$ of the expected response of the two control groups will be conducted. Let $\alpha _{Ept}$ denote the respective significance level of the pre-test and $z_{1-\alpha _{Ept}}$ denote the $1-\alpha _{Ept}$-quantile of the standard normal distribution. The corresponding hypothesis $H_0^{Ept}: | \mu _C - \mu _H | \ge \Delta$ versus $H_1^{Ept}: | \mu _C - \mu _H | < \Delta$ is tested using

1 $$\begin{aligned} Z_{Ept}=\frac{\vert \bar{y}_C - \bar{y}_H \vert - \Delta }{\sqrt{\frac{1}{n_C}+\frac{1}{n_H}}} < - z_{1-\frac{\alpha _{Ept}}{2}} {\; ,} \text { with } \Delta >0 \end{aligned}$$

or using the two one-sided shifted test statistics [27]. Note that $var(\bar{y}_H - \bar{y}_C) = {1}/{n_H}+{1}/{n_C}$ as there are $n_H$ and $n_C$ observations in the groups.

#### Superiority test with historical controls

If the equivalence pre-test rejects $H_0^{Ept}$, the comparability of the randomized and historical controls is established. In this case, the superiority of the response data of the $n_{E}$ patients of the experimental group will be compared to the pooled $n_{C}+ n_H$ control data. This is done by testing the superiority of *E* versus *C*+*H* with the hypothesis $H_0^{S_1}: \mu _E - [\omega \mu _H + (1-\omega )\mu _C] \le 0$ versus $H_1^{S_1}: \mu _E - [\omega \mu _H + (1-\omega )\mu _C] > 0$ at the significance level $\alpha _{S_1}$. Note, given $\mu _H=\mu _C$ this results in the original superiority hypothesis of *E* versus *C*. But given $|\mu _H-\mu _C|<\Delta$ from the equivalence test this refers to non-inferiority between the groups *E* and *C*. To test $H_0^{S_1}$ versus $H_1^{S_1}$, we propose to use the weighted test statistic

2 $$\begin{aligned} Z_{S_1} = \frac{ \bar{y}_E - \left( \omega \cdot \bar{y}_H + ( 1- \omega ) \cdot \bar{y}_C \right) }{ \sqrt{\frac{1}{n_{E}}+ \frac{\omega ^2 }{n_{H}} + \frac{(1-\omega )^2 }{n_{C}} }} \end{aligned}$$

where $\omega \in [0,1]$ indicates the weight of the historical control data in the estimate of the treatment effect. Note, the variance of the sample estimate

$$\begin{aligned} var(\omega \cdot \bar{y}_H + ( 1- \omega ) \cdot \bar{y}_C) = \frac{\omega ^2 }{n_{H}} + \frac{(1-\omega )^2 }{n_{C}} \end{aligned}$$

is minimized for $\omega ^*= n_{H} / (n_{H}+n_{C})$ (see Appendix A equation (A1)), which corresponds to a simple pooling of historical and concurrent controls with respect to their sample size. We will use the optimal weight $\omega ^*$ in the following considerations, which results in $var(\omega \cdot \bar{y}_H + ( 1- \omega ) \cdot \bar{y}_C) = \frac{1 }{n_{H} +n_{C}}$ as well as

$$\begin{aligned} Z^*_{S_1} = \frac{ \bar{y}_E - \left( \frac{n_{H}}{ n_{H}+n_{C}} \cdot \bar{y}_H + \frac{n_{C}}{ n_{H}+n_{C}} \cdot \bar{y}_C \right) }{ \sqrt{\frac{1}{n_{E}} + \frac{1}{ n_{H}+n_{C}} }} \end{aligned}$$

for the superiority test ($S_1$). As ${n_{H}}/({ n_{H}+n_{C}}) \cdot \bar{y}_H + {n_{C}}/({ n_{H}+n_{C}}) \cdot \bar{y}_C$ is just the overall mean of the responses of the historical and randomized controls, the test statistic $Z^*_{S_1}$ is the usual z-test for comparing two means.

#### Superiority test without historical controls

If the equivalence pre-test does not reject $H_0^{Ept}$, the comparability of the control groups is not established. Then, additional $n_{E}'$ and $n_{C}'$ patients are recruited in the treatment and control groups respectively. This leads to the final superiority test ($S_2$) which is based on the pooled data of $N_E=n_{E} + n_{E}'$ patients randomly allocated to the experimental treatment compared to $N_C=n_{C} + n_{C}'$ patients randomly allocated to the control treatment. Then the superiority hypothesis $H_0^{S_2}: \mu _E - \mu _C \le 0$ versus $H_1^{S_2}: \mu _E - \mu _C > 0$ is tested at the significance level $\alpha _{S_2}$ using the test statistic

3 $$\begin{aligned} Z_{S_2} = \frac{ \bar{y}'_E - \bar{y}'_C }{ \sqrt{\frac{1}{n_{E} + n_{E}'}+\frac{1}{n_{C} + n_{C}'}}}. \end{aligned}$$

Let $\varphi ^{\alpha _{Ept}}_{Ept}, \varphi ^{\alpha _{S_1}}_{S_1}$ and $\varphi ^{\alpha _{S_2}}_{S_2}$ denote the respective decision functions of the z-tests (1), (2) and (3) taking the value 1 for the rejection of the respective null hypothesis and 0 otherwise. Altogether the decision function of the multiple test of the hypotheses of the superiority tests ($S_1$) and ($S_2$) of the *Fill-it-up*-design can be written as

$$\begin{aligned} \psi _{FIU}=\max \left( \varphi ^{\alpha _{Ept}}_{Ept}\varphi ^{\alpha _{S_1}}_{S_1},(1- \varphi ^{\alpha _{Ept}}_{Ept})\varphi ^{\alpha _{S_2}}_{S_2}\right) . \end{aligned}$$

In particular, the appropriate choice for the significance levels $\alpha _{S_1}$, $\alpha _{{S_2}}$ and $\alpha _{Ept}$ of the three tests to control the family wise error rate will be discussed below.

## Evaluation of the Fill-it-up-design

Below we investigate:

1. How to split the sample size of the randomized trial into the two steps of the *Fill-it-up*-design?
2. What are the possible choices for the equivalence margin $\Delta$ and how are the tests related?
3. How can the type 1 error rate for the *Fill-it-up*-design be controlled? Here we determine significance levels for the tests ($S_1$), ($S_2$) and (*Ept*) such that the whole procedure controls the type I error rate at 0.05.
4. What is the power of the *Fill-it-up*-design compared to a single step trial showing the same effect with a comparable level of evidence using only ($S_2$)?

### Allocation of sample sizes between the stages of the Fill-it-up-design

With regard to the sample size determination, a major question would be to decide, about the fraction $\gamma$ of patients allocated in the first step. Of course, one would require that either the test ($S_1$) or the test ($S_2$) should have a specific power to detect the treatment effect of $\delta$. To be more specific, denote $N_E=n_E+n'_E$ and $N_C=n_C+n'_C$ and $\gamma N_E= n_E, \gamma N_C= n_C.$ The superiority test ($S_1$) is conducted with the sample sizes $n_C$ and $n_E$ in the randomized first step and a sample of the size $n_H$ historical controls fulfilling

4 $$\begin{aligned} \frac{1}{\delta ^2} \left( z_{1-\alpha _{{S_1}}} + z_{1-\beta _{{S_1}}} \right) ^2 = \frac{n_E \cdot (n_H + n_C)}{n_E + n_H + n_C} = \frac{\gamma N_E \cdot (n_H + \gamma N_C)}{\gamma (N_E + N_C) + n_H }, \end{aligned}$$

to achieve a power of $1-\beta _{{S_1}}$ of the superiority test ($S_1$) not accounting for the pre-test. As $N_E+N_C =n_E+n'_E + n'_C+n_C$ is planned in advance, for the test ($S_2$) to test the hypothesis $H_0^{S_2}: \mu _E - \mu _C \le 0$ versus ${\tilde{H}}_1^{S_2}: \mu _E - \mu _C = \delta >0$ the sample size can be determined by [15]

5 $$\begin{aligned} \frac{N_E \cdot N_C}{N_E + N_C} = \frac{1}{\delta ^2} \left( z_{1-\alpha _{{S_2}}} + z_{1-\beta _{{S_2}}} \right) ^2{\; ,} \end{aligned}$$

to achieve a power of $1-\beta _{{S_2}}$ of the superiority test ($S_2$) ignoring the equivalence pre-test. Substitution for $\delta$ from (4) in (5) results in

$$\begin{aligned} \frac{N_E + N_C}{N_E \cdot N_C} \cdot \frac{\gamma N_E \cdot (n_H + \gamma N_C)}{\gamma (N_E + N_C) + n_H } = \left( \frac{ z_{1-\alpha _{{S_1}}} + z_{1-\beta _{{S_1}}}}{z_{1-\alpha _{{S_2}}} + z_{1-\beta _{{S_2}}} }\right) ^2 {\; ,} \end{aligned}$$

a quadratic equation in $\gamma$ with the solutions:

$$\begin{aligned} \gamma & = \frac{1}{2} \left( - \frac{n_H}{N_C} + \left( \frac{ z_{1-\alpha _{{S_1}}} + z_{1-\beta _{{S_1}}}}{z_{1-\alpha _{{S_2}}} + z_{1-\beta _{{S_2}}} }\right) ^2 \pm \sqrt{ \left( \frac{n_H}{N_C} - \left( \frac{ z_{1-\alpha _{{S_1}}} + z_{1-\beta _{{S_1}}}}{z_{1-\alpha _{{S_2}}} + z_{1-\beta _{{S_2}}} }\right) ^2 \right) ^2 + \frac{4 n_H}{N_E+N_C} \cdot \left( \frac{ z_{1-\alpha _{{S_1}}} + z_{1-\beta _{{S_1}}}}{z_{1-\alpha _{{S_2}}} + z_{1-\beta _{{S_2}}} }\right) ^2} \right) . \end{aligned}$$

In applications, one might favor the special setting of equal type I error $\alpha _{{S_1}}=\alpha _{{S_2}}$ and type II error $\beta _{{S_1}}=\beta _{{S_2}}$ probabilities, which result in

$$\begin{aligned} \frac{ z_{1-\alpha _{{S_1}}} + z_{1-\beta _{{S_1}}}}{z_{1-\alpha _{{S_2}}} + z_{1-\beta _{{S_2}}} } = 1 \end{aligned}$$

as well as balanced sample sizes $N_E=N_C=N$ so that the expression simplifies to

6 $$\begin{aligned} \gamma = \frac{1}{2N} \left( N - n_H + \sqrt{ N^2 + n_H^2} \right) {\; ,} \end{aligned}$$

which is the only positive solution for $\gamma$. It should be noted that $\gamma$ decreases with increasing $n_H$ with a maximal value of $\gamma =1$ in case $n_H$ is zero. Further with $N^2 + n_H^2 \ge n_H^2$ it results that $\gamma \ge 1/2.$ So at least 50% of the total sample size is necessary in the first step randomized trial. Choices of $\gamma$ are described in the sections entitled “Power of the Fill-it-up-design” and “The Fill-it-up-design in practice”.

Consequently, we note that for the *Fill-it-up*-design, the total sample size is $N_{FIU}=N_E+N_C$ if the null hypothesis of the pre-test is not rejected and the historical controls are rejected. In the situation that the null hypothesis of the pre-test can be rejected, the resulting sample size is $\gamma N_{FIU}$ for the newly recruited patients in addition to the $n_H$ historical controls. The maximum sample size of newly recruited patients of the design can therefore be characterized as $N_{FIU}$.

### Relation between equivalence test and superiority test

To elaborate on the relation between the equivalence margin $\Delta$ and the effect size $\delta$ we consider the case where the data of the historical controls are used in the efficacy evaluation using the superiority test ($S_1$). The evaluation of efficacy from the randomized data only i.e. application of superiority test ($S_2$), seems to be unrelated to the equivalence margin $\Delta$.

When applying the superiority test ($S_1$), the *weighted effect size* taking $\delta = \mu _E - \mu _C$ can be rewritten as $t = \mu _E - \left( \omega ^* \cdot \mu _H + ( 1- \omega ^*) \cdot \mu _C \right) = \mu _E - \mu _C + \omega ^* (\mu _C - \mu _H) = \delta + \omega ^* (\mu _C - \mu _H)$. For simplicity, assume that larger expected responses are associated with an improved response to treatment and that the randomized control group shows a smaller or equal effect than the experimental group $\mu _E \ge \mu _C$. Depending on the expected difference between the historical control group and the randomized control group we consider two cases.

Firstly we assume that the expected response of the historical control group is larger than the corresponding response of the randomized control group, i.e. $\mu _C - \mu _H = - \Delta ^* \le 0$. In this case, the weighted effect size $t$ is smaller than the effect size $\delta = \mu _E - \mu _C$, i.e. $t = \delta - \omega ^*\Delta ^* \le \delta .$ In this case, the response under control is overestimated, which plays against the target to establish a positive treatment effect using the historical controls, because a potential treatment difference $\delta$ between (pooled) control and experimental group is likely to be overlooked. So one might favor only a small equivalence margin $\Delta$ to preserve as much as possible from the true treatment effect $\delta$. However, a small $\Delta$ might result in a lack of power for the equivalence test.

Second, consider the case $\mu _C - \mu _H = \Delta ^* \ge 0$ such that the expected response of the historical control group is smaller than the corresponding response of the randomized control group. This leads to $t = \delta + \omega ^*\Delta ^* \ge \delta$. Thus, the expected response of the combined control group is reduced by the historical controls, increasing the treatment effect to be detected for the experimental group. In this case, the response under control is underestimated, which is a rather liberal situation. This should be avoided, as the superiority test ($S_1$) is powered for a smaller effect resulting in the risk of an uncontrolled erroneous decision for a positive treatment effect even if the actual treatment effect is 0. To control this situation, the equivalence margin $\Delta$ has to be small enough, to reflect the maximal tolerated deviation (inferiority of expected response of the historical from the randomized control group) on the one hand but also has to be carefully chosen to maintain the expected treatment difference $\delta$ between control and experimental group.

Furthermore $\Delta <\delta$ should be applied as the upper limit of the equivalence margin $\Delta$ to ensure that the control groups do not differ by more than the treatment effect for which the study is powered. Additionally, as stated earlier [27] there exists a lower limit for the equivalence margin regarding the rejection region of the two one sided tests. If $\sqrt{\frac{1}{n_H}+\frac{1}{n_C}} > \frac{\Delta }{z_{1-\alpha _{Ept}}}$ applies, the null hypothesis of the equivalence pre-test will never be rejected. To summarize the choice for $\Delta$ is in any case restricted to

7 $$\begin{aligned} \sqrt{\frac{1}{n_H}+\frac{1}{n_C}} \cdot z_{1-\alpha _{Ept}} \le \Delta < \delta . \end{aligned}$$

In summary, the equivalence pre-test does not only protect against a potentially enlarged treatment difference between pooled control groups and the experimental group but also against the case that the potential treatment effect between randomized control and experimental groups is reduced.

### Type I error probability of the Fill-it-up-design

Next, the overall test size $\alpha$ is evaluated including the levels of the individual tests $\alpha _{Ept}, \alpha _{S_1}$ and $\alpha _{S_2}$. To determine the joint distributions we observe from (1), (2) and (3) by direct calculation that the expectation of $Z_{Ept}$ results in $\mu _{Ept}:=\left( \vert \mu _C - \mu _H \vert - \Delta \right) /{\sqrt{\frac{1}{n_H}+\frac{1}{n_C}}}$ with corresponding variance $\sigma _{Ept}:=Var (Z_{Ept}) = 1$.

The expectations for the two superiority test statistics $Z_{S_1}$ and $Z_{S_2}$ yield $\mu _{S_1}:=\left( \mu _E - \left[ \omega ^* \cdot \mu _H + ( 1- \omega ^* ) \cdot \mu _C \right] \right) / \sqrt{\frac{1}{n_{E}}+\frac{1}{n_C+n_H}}$ and $\mu _{S_2}:=\left( \mu _E - \mu _C \right) / \sqrt{\frac{1}{n_{E}+n'_{E} }+\frac{1}{n_{C}+ n'_C}}$. The corresponding variance of $Z_{S1}$ is

8 $$\begin{aligned} \sigma _{S_1}:=Var (Z_{S_1}) & = \left( \frac{1}{n_{E}}+\frac{1}{n_C+n_H}\right) ^{-1} \left( \frac{1}{n_E}+ \frac{\omega ^{2}}{n_H}+\frac{(1-\omega )^2}{n_C} \right) \nonumber \\ & = \frac{n_E ( n_C +n_H)}{n_C + n_H + n_E} \cdot \frac{n_C n_H + \omega n_E n_C + (1-\omega )^2 n_E n_H}{n_E n_H n_C}. \end{aligned}$$

In the special case where $\omega = \omega ^* = n_H/(n_H + n_C)$ we obtain $\sigma _{{S_1}^*}:=Var (Z_{S_1}^*)=1$. Similarly, we obtain $\sigma _{S_2}:=Var (Z_{S_2}) =1.$ It should be noted that with $\omega = \omega ^*$, $Z_{S_1}$ is uncorrelated to $Z_{Ept}$ (see Appedix A equation (A2)). On the other hand, $Z_{S_2}$ and $Z_{Ept}$ are correlated (see Appendix A equation (A3)). From this expression, it follows that, as the sample size of the historical control increases and the allocation to groups *C* and *E* is balanced (i.e., $n_E+n'_E = n_C+n'_C$), the covariance between the test statistic $Z_{S_2}$ and $Z_{Ept}$ approaches 0.5. This occurs if an equal number of patients are allocated to the control group *C* in both steps of the trial (i.e., $n_C=n'_C$). The covariance is at most $1/\sqrt{2}$ when $n'_C$ becomes very small. If, however $n'_C$ becomes large, $Cov ( Z_{S_2},Z_{Ept} )$ decreases to zero and the tests become uncorrelated.

Formulas (8) and Appendix A equations (A2) and (A3) can be used to calculate the type I error probabilities from the respective joint distributions. The overall type I error probability of the procedure should satisfy

$$\begin{aligned} E\left( \psi _{FIU}\right) \le \alpha \end{aligned}$$

and can be obtained by

9 $$\begin{aligned} E\left( \psi _{FIU}\right) =\underbrace{E\left( \varphi ^{\alpha _{Ept}}_{Ept}\varphi ^{\alpha _{S_1}}_{S_1}\right) }_{:=\alpha _{Ept,S_1}}+\underbrace{E\left( \left( 1- \varphi ^{\alpha _{Ept}}_{Ept}\right) \varphi ^{\alpha _{S_2}}_{S_2}\right) }_{:=\alpha _{Ept^c,S_2}}. \end{aligned}$$

**Fig. 2:**
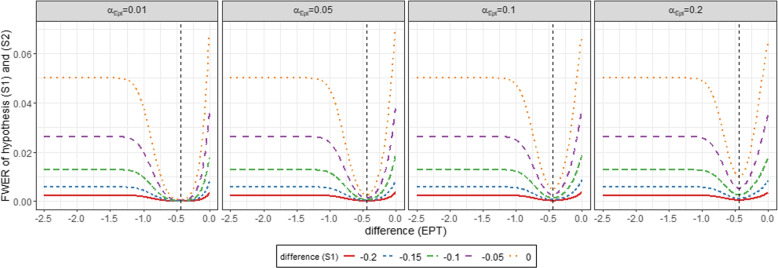
Family wise error rate testing simultaneously superiority tests ($S_1$) and ($S_2$) for different scenarios of the Fill-it-up-design depending on the choice of the significance level of the equivalence pre-test. A medium effect size $\delta =0.5$ with $n_H=500$ historical controls and an equivalence margin of $\Delta =0.44$ is examined

**Fig. 3:**
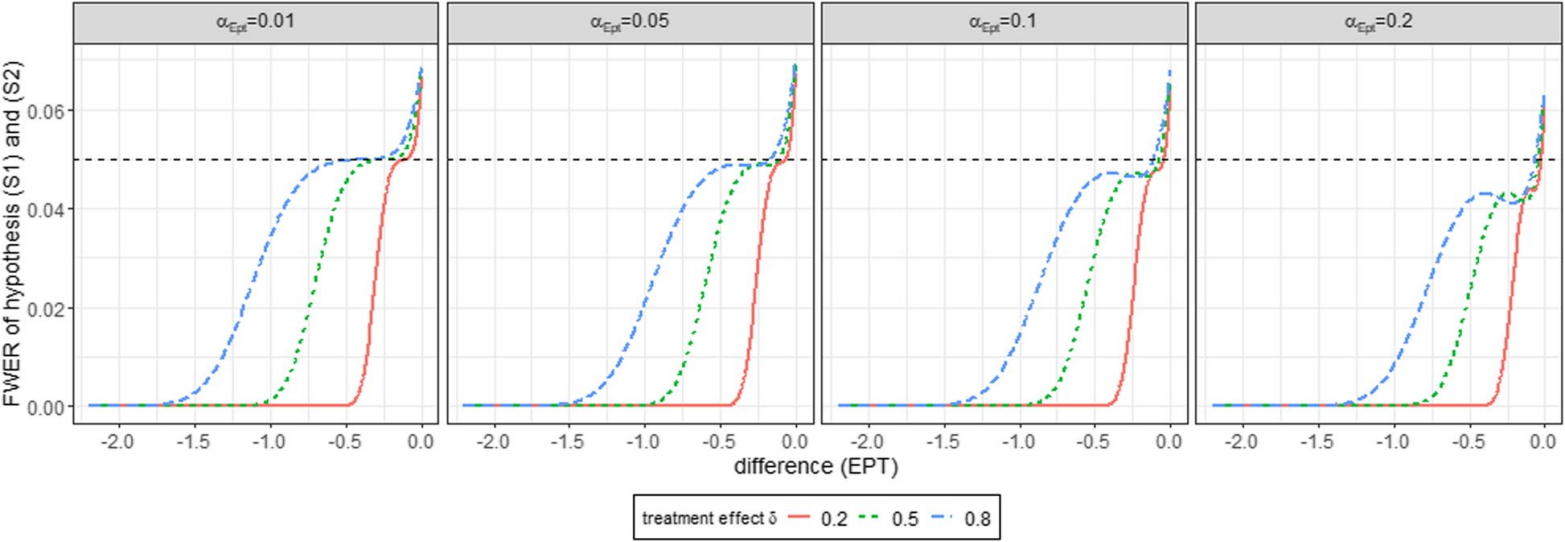
Family wise error rate testing simultaneously superiority tests ($S_1$) and ($S_2$) assuming $\mu _E=\mu _C=0$ depending on the choice of the significance level of the equivalence pre-test. $n_H=500$ historical controls were included considering a small effect $\delta =0.2$ with equivalence margin $\Delta =0.19$, medium effect $\delta =0.5$ with equivalence margin $\Delta =0.44$ and large effect $\delta =0.8$ with equivalence margin $\Delta =0.70$

For this purpose, the combinations of the pre-test and the superiority tests (*S*1) and (*S*2) are summarised. Superiority test (*S*1) is performed if the null hypothesis of the pre-test is rejected ($\varphi ^{\alpha _{Ept}}_{Ept}=1$), so that the expected value is determined via the product of the decision functions $\varphi ^{\alpha _{Ept}}_{Ept}$ and $\varphi ^{\alpha _{S_1}}_{S_1}$. If the null hypothesis of the pre-test is not rejected ($\varphi ^{\alpha _{Ept}}_{Ept}=0$), the superiority test (*S*2) is performed so that the expected value is determined via the product of $1-\varphi ^{\alpha _{Ept}}_{Ept}$ and $\varphi ^{\alpha _{S_2}}_{S_2}$. The formulas for the calculation of $\alpha _{Ept,S_1}$ and $\alpha _{Ept^c,S_2}$ using normal densities can be found in Appendix B. As discussed in Viele et al. [30] relaxing the significance level of the equivalence pre-test (*Ept*), would decrease the average sample size of the randomized trial while using the historical controls. Similar to him we therefore consider $\alpha _{Ept} \in \{0.01, 0.05, 0.1, 0.2\}$ and calculate equation (9) using Appendix B equations (B1) and (B2) numerically for various settings to obtain the maximum type I error probability. All computations are conducted with R (R Core Team, 2018) [22], version 4.1.2 for Windows (64 bit). The calculation of the integrals for the multivariable normal distribution displayed in Appendix B equations (B1) and (B2) was calculated numerically using the R package *“mvtnorm”*. For this purpose, the expected response of the control group was fixed at 0 and the expected response of the historical control group was varied between -2.5 and 0 according to the null hypothesis $H_0^{Ept}$ to illustrate the difference of the control groups $\bar{y}_H - \bar{y}_C$. The expected response of the experimental group was chosen as a function of the expected response of the historical control group such that the difference $\bar{y}_E-(\omega \bar{y}_H+(1-\omega )\bar{y}_C)$ of $\lbrace -0.2, -0.15, -0.1, -0.05, 0 \rbrace$ is investigated according to the null hypothesis $H_0^{S_1}$. Figure 2 illustrates the family wise error rate depending on the underlying difference between the expected response of the historical and current controls ($\bar{y}_H - \bar{y}_C$, difference (*Ept*)) as well as underlying weighted treatment effect of the superiority test ($\bar{y}_E-(\omega \bar{y}_H+(1-\omega )\bar{y}_C)$, difference ($S_1$)) for the four different choices of the significance level of the equivalence pre-test. Figure 2 shows that under the respective null hypotheses $H_0^{Ept}$ and $H_0^{S_1}$ (i.e. lines left to the dashed vertical line), the family wise error rate is maximized for larger differences between the expected response of historical and current controls (difference (*Ept*)) together with a weighted treatment effect (difference ($S_1$)) of zero. Under the respective null hypotheses $H_0^{Ept}$ and $H_0^{S_1}$ the maximum family wise error rate is kept at $5\%$. According to equation (7) for the calculation of the family wise error rate considering a medium effect size, the equivalence margin $\Delta$ was set to 0.44. Similar results are obtained for the settings for small ($\delta =0.2$, $\Delta =0.19$) in Appendix C figure C1 and large ($\delta =0.8$, $\Delta =0.70$) in figure C2 respectively. We also obtain the same results for other choices of the margin. In these cases, there is a shift in the curves, whereby the minimum is always achieved when the difference of the equivalence pre-tests (difference (*Ept*)) corresponds to the equivalence margin. The family wise error rate also maximizes in these cases, the further the difference of the equivalence pre-tests deviates from the selected margin. Figure 3 displays a special case in which both the expected responses of the control group $\mu _H$ and that of the experimental group $\mu _E$ are set equal to 0. The difference between historical and randomized controls is indicated on the x axis. The various lines reflect the approximation of the integrals from equation (9) depending on the different settings for a small, medium and large treatment effect $\delta$. For this investigation we used the same choices for the equivalence margin as before, i.e. $\Delta =0.19$ for a small, $\Delta =0.44$ for a medium and $\Delta =0.7$ for a large treatment effect.

### Power of the Fill-it-up-design

Next, the power of the procedure to detect a treatment effect of $\delta =\mu _E - \mu _C$ is evaluated. Recall that the statistical test ($S_2$) is planned with a power of $1-\beta _{S_2}$ and similarly the test $S_1$ should show a power of at least $1-\beta _{S_1}=1-\beta _{S_2}$.

It is worth noting that an anticipated effect of $\delta$ for the difference in means together with an confirmed equivalence between historical and randomized controls $\vert \mu _H - \mu _C \vert \le \Delta$ results in the expected weighted treatment difference of $\mu _E - \left( \omega ^{*} \cdot \mu _H + ( 1- \omega ^{*}) \cdot \mu _C \right) = \mu _E - \mu _C - \omega ^{*} (\mu _H - \mu _C ) > \delta - \frac{n_H}{n_H+n_C} \Delta .$ This expression almost vanishes with small $n_C$ if $\Delta$ is in the same magnitude as $\delta$, i.e. the anticipated effect $\delta$ is overlooked. From the power perspective, this means, that for $\delta < \frac{n_H}{n_H+n_C} \Delta$ most of the trials will continue to the whole sample size and with $\delta > \frac{n_H}{n_H+n_C} \Delta$ some trials use historical control data. Similar to equation (9) for the overall power of the *Fill-it-up*-design $(1-\beta _{FIU})$ of the test assuming the effect of $\delta = \mu _E - \mu _C$ using the anticipated decision rule it holds

10 $$\begin{aligned} 1-\beta _{FIU} = 1 - \left( \beta _{Ept,S_1} + \beta _{Ept^c,S_2} \right) . \end{aligned}$$

Here $\beta _{Ept, S_1}$ is the type II error probability for detecting equivalence in the pre-test without proving the difference in the superiority test (*S*1) and $\beta _{Ept^c, S_2}$ is the type II error probability for not detecting equivalence in the equivalence pre-test and not proving the difference. The formulas for the calculation of $\beta _{Ept,S_1}$ and $\beta _{Ept^c,S_2}$ using normal densities can be found in Appendix D. The equations show, that one has to pay a price for the extension implemented in the *Fill-it-up*-design compared to a single trial using only the ($S_2$) test. If both type II error probabilities $\beta _{Ept, S1}$ and $\beta _{Ept^c, S2}$ could be set up to 0.2, then the overall power does not exceed 0.6. One may ask for a combination of the type II error probabilities $\beta _{Ept, S1}$ and $\beta _{Ept^c, S2}$ so that the overall power $1-\beta _{FIU}$ is about 0.8. We evaluate this and compare the total sample size of the *Fill-it-up*-design $N_{FIU}=N_E+N_C$ as well as the reduced sample size $\gamma N_{FIU}$ and the average sample size $AVN=\gamma N_{FIU}+ (1-\alpha _{Ept}) (N_{FIU}-\gamma N_{FIU})$ with a sample size of a corresponding one step design using the ($S_2$) test only. The calculation of the overall power of the design in equation (10) was implemented using the integrals of multivariable normal distributions of Appendix D equations (D1) and (D2) and was calculated numerically using the R package *“mvtnorm”*. Table 1 shows possible choices of the power of the superiority tests $(S_1)$ and $(S_2)$ in column “$1-\beta _{S_1}=1-\beta _{S_2}$” to keep the overall power of the *Fill-it-up*-design at a minimum of $80\%$ depending on the choice of the significance level of the equivalence pre-test and the equivalence margin $\Delta$. As Table 1 shows, we can maintain the overall power of the *Fill-it-up*-design at 0.8. For this purpose we need to adjust the corresponding type II error probabilities of the two superiority tests ($S_1$) and ($S_2$) for the different scenarios of significance levels and the equivalence margin. With increasing equivalence margin $\Delta$ the type II error probabilities $\beta _{S_1}=\beta _{S_2}$ have to be chosen smaller resulting in larger sample sizes for the two steps of the *Fill-it-up*-design. Similar results are obtained for small and large treatment effects as displayed in Appendix E Tables E1 and E2 respectively. Compared to this the one step design using the $(S_2)$ test only for power $1-\beta _{S_2}=0.8$ keeping the significance level $\alpha _{S_2}$ at $5\%$ requires the sample sizes displayed in Table 2 for the considered treatment effects $\delta$. Tables 1 and 2 show that if we can show the equivalence of the control groups, we can save a certain amount of the sample size. For example, we can save 52 subjects for the significance level $\alpha _{Ept}=0.05$ of the equivalence test with a medium treatment effect comparing $\gamma N_{FIU}=60$ and $N_{FIU}=112$. Compared to the ($S_2$) test alone, we can save 40 subjects here. However, if we compare the maximum sample size of the design $N_{FIU}=112$ with $N_{S_2}=100$, it becomes clear that we have a premium to pay if the null hypothesis of the equivalence pre-test is not rejected. In this case, 12 more subjects are needed. For the highest significance level of $\alpha _{Ept}=0.2$, $28-46$ subjects can be saved, if equivalence can be shown. However, if we are not able to prove equivalence, we have to recruit additional subjects. In the latter case, the design requires $2-38$ additional subjects compared to the required sample size when using a design with a single superiority test ($S_2$ in our notation) only. We can also observe this from the average sample size. It tends to be able to keep up with the one step method for the smaller choices of the equivalence margin. We also observe similar effects for small and large treatment effects. Here, Appendix E Tables E1 and E2 present the respective cases in comparison to the one step case from Table 2 ignoring the historical controls. Overall, it can be seen that the maximum sample size of the *Fill-it-up*-design is larger than or equal to that of the single stage model if the historical controls are not taken into account. In particular, the maximum sample size increases the more heterogeneous the two control groups are. Concerning the proportion of patients who are required in the first step of the design, we take a look at the value of $\gamma$ in the present scenarios. If we look at the columns $N_{FIU}$ and $\gamma N_{FIU}$ of Table 1, it becomes clear that the proportion of patients in step 1 ranges between 53$\%$ and 54$\%$ of the total needed sample size of both steps in all cases. As mentioned in the section entitled “Allocation of sample sizes between the stages of the Fill-it-up-design”, $\gamma$ is greater than $50\%$ in any case and is therefore within a range in which only slightly more than half of the patients need to be recruited in the first step. Similar results can be obtained for small and large treatment effects displayed in Appendix E Tables E1 and E2. For small treatment effects, the values for $\gamma$ are in the range of 64$\%$ to 68$\%$ and thus higher than those of the medium treatment effect. For large treatment effects, on the other hand, lower values are observed, ranging between 51$\%$ and 55$\%$. It can therefore be concluded that smaller treatment effects lead to a proportionally higher number of patients having to be recruited in the first step than in the case of larger treatment effects. As a result, fewer patients can potentially be saved, especially in these cases.

**Table 1:** Overall power and sample sizes for different scenarios of the Fill-it-up-design depending on the choice of the equivalence margin $\Delta$ considering a medium effect size $\delta =0.5$ including $n_H=500$ historical controls and significance levels $\alpha _{S_1}=\alpha _{S_2}=0.05$

$\alpha _{Ept}$	$\Delta$	$N_{FIU}$	$\gamma N_{FIU}$	*AVN*	$1-\beta _{S_1}=1-\beta _{S_2}$	$1-\beta _{Ept,S_1}$	$1-\beta _{Ept^c,S_2}$	$1-\beta _{FIU}$
0.01	0.4596	100	54	100	0.80	0.9530	0.8471	0.8001
0.01	0.4798	100	54	100	0.80	0.9871	0.8130	0.8001
0.05	0.3250	102	54	100	0.81	0.9596	0.8403	0.8000
0.05	0.3901	138	74	136	0.90	0.9608	0.8392	0.8000
0.10	0.2303	124	66	120	0.87	0.9637	0.8368	0.8005
0.10	0.3652	124	66	120	0.87	0.8442	0.9558	0.8000
0.20	0.1663	102	54	94	0.81	0.9722	0.8279	0.8001
0.20	0.3331	102	54	94	0.81	0.9403	0.8599	0.8002

$\alpha _{Ept}$ Two-sided significance level of equivalence pre-test, $N_{FIU}$ Maximum sample size of the Fill-it-up-design, $\gamma N_{FIU}$ Sample size of the first stage of the Fill-it-up-design, $AVN$ Average sample size, $\beta _{S_1}$ Type II Error Probability superiority test (*S*1), $\beta _{Ept,S_1}$ Type II Error Probability of equivalence pre-test and superiority test (*S*1), $\beta _{Ept^c,S_2}$ Type II Error Probability of equivalence pre-test and superiority test (*S*2), $1-\beta _{FIU}$ Power of the Fill-it-up-design

**Table 2:** Sample sizes for different scenarios of the superiority test ($S_2$) without historical controls considering small, medium and large effect sizes $\delta$ with a Power of $1-\beta _{S_2}=0.8$ and for the significance level $\alpha _{S_2}=5\%$

$\delta$	$N_{S_2}$
0.2	620
0.5	100
0.8	40

## Comparison with Bayesian approach

As mentioned in the beginning, many Bayesian approaches to the use of historical controls have already been evaluated. To compare our method with the existing ones, we consider the meta-analytic predictive priors approach [19]. As already evaluated, the robust MAP priors offer a good method that we would like to use for comparison concerning family wise error rate and power of the designs. For the comparison of the previous evaluations of the *Fill-it-up*-design with the MAP approach, we use the R package RBesT [31]. We again assume 500 historical controls and calculate the required number of patients for the first step analogously to the *Fill-it-up*-design. For the evaluation of the MAP approach, we will consider the full data set at the end, which in the *Fill-it-up*-design corresponds to the case in which the equivalence test cannot reject the null hypothesis. In the following, we will refer to this sample as “full-sample”. In addition, we will also calculate the type I error probability and power for the case in which no further recruitment takes place to ensure the best possible comparability of the two methods. This sample will be referred to as “sub-sample” in the following. In Bayesian approaches, modeling heterogeneity between historical controls is of central importance. Since we start from only one historical study, the choice here needs to be especially careful. We address this by comparing three different settings for heterogeneity. First, we assume a half normal distribution with an expected value of 0 and variance $\frac{\sigma _{h}}{2}$. As further settings, we have chosen truncated normal and truncated cauchy distributions with the same expected values and variances. Typically, one would estimate the variance with $\sigma$, but here we would like to use the more conservative setting $\frac{\sigma }{2}$. As recommended, we use the robust MAP approach described previously [26]. As described by Roychoudhury et al. [25] we transfer the previously defined frequentist hypotheses of the superiority test $S_2$ into a dual-criterion Bayesian design. We assume the following decision functions:

$$\begin{aligned} P \left( y_E - y_C> 0 \right)> 0.95 \quad \text {and} \quad P \left( y_E - y_C> \delta - 0.2 \right) > 0.5 \end{aligned}$$

**Fig. 4:**
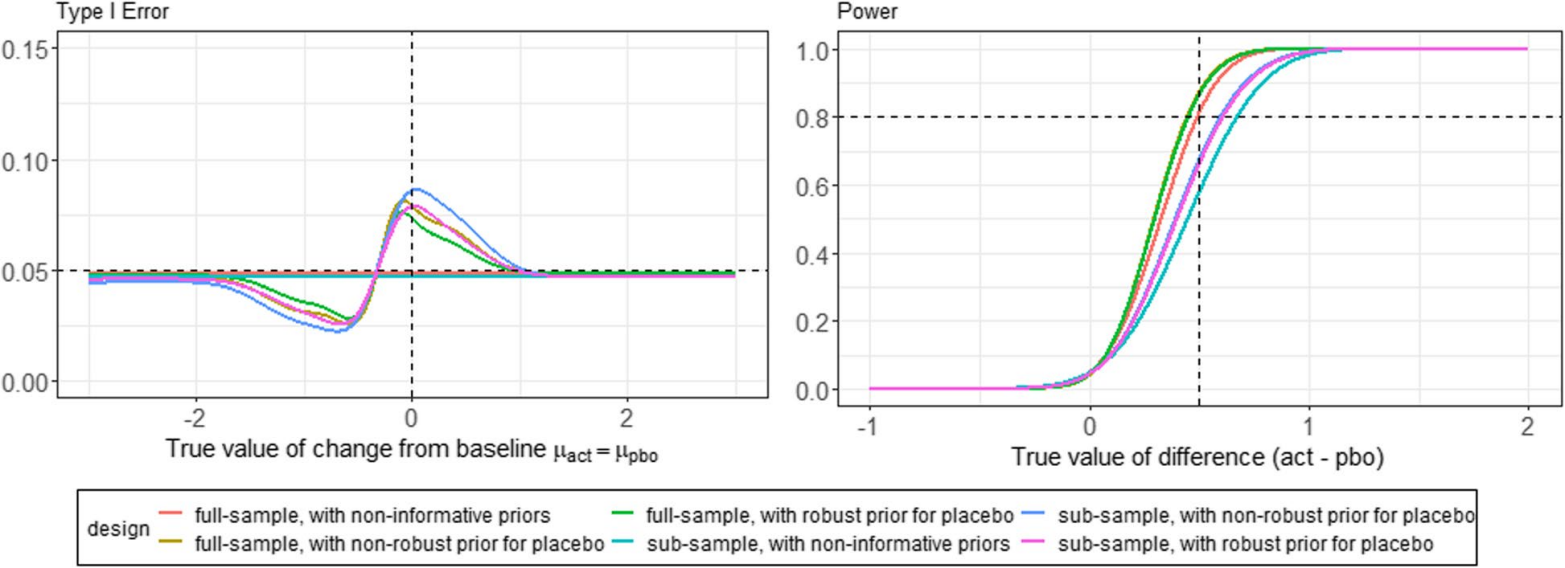
Evaluation of type I error and power for MAP approach for full-sample and sub-sample using non-informative, non-robust and robust priors and half normal distribution for heterogeneity

This choice implies that the design accounts for both statistical significance and clinical relevance. A decision value (DV) of $\delta - 0.2$ was chosen, as this corresponds to the choice of the treatment effect to be detected of 0.5. This can also be seen in the power graphs Fig. 4 and Appendix F figures F1 and F2. The DV of $\delta - 0.2$ achieves a power of $50\%$, which by no means indicates underpowering according to Roychoudhury et al. [25]. As can be seen, the desired power of $80\%$ is achieved with the expected treatment effect of 0.5. This allows the study settings to be comparable between the frequentist *Fill-it-up*-design and the bayesian robust MAP approach. For the evaluation of the MAP approach, the same framework conditions were set as previously in the *Fill-it-up*-design. For the displayed evaluations we again assumed a medium treatment effect of 0.5 to be detected and calculated the underlying sample size in the same way as in the *Fill-it-up*-design, using the Eqs. (5) and (6). We have assumed $\beta =0.2$ and $\alpha =0.05$ for this purpose, following the previous sections and evaluations. As possible scenarios, reflected by the different lines in Fig. 4, we formed the type I error and power among non-informative priors, non-robust priors and robust priors for the placebo, ie. control group based on the prior information of the historical controls. Furthermore, we can see the different courses for the full- and sub-sample assumption. Figure 4 shows that the type I error of the MAP approach for a negative difference $\mu _E-\mu _C$ initially complies well with the 5$\%$. The closer the difference approaches zero, the lower the type I error becomes at first. However, there is an increase in the type I error, which reaches its maximum when the expected responses of the two newly randomized groups *E* and *C* are equal. In this range, a type I error inflation to approx. 7.5$\%$ is possible. Similar results are obtained using the truncated normal distribution in Appendix F figure F1 and the truncated cauchy distribution in Appendix F figure F2. For the truncated cauchy distribution, however, a type I error inflation can also be observed at approximately $\mu _E-\mu _C <-2$. Under all three distribution assumptions, it should be noted that the choice of the non-informative prior allows compliance with the 5$\%$ in both the full and the sub-sample. For the power, we obtain similar results. As expected, smaller treatment effects than the planned one are detected only with low power speaking of a true value of the difference between *E* and *C* of below 0.5. However, the power increases steadily and exceeds the 80$\%$ threshold for most scenarios when the desired treatment effect is reached. An exception are the designs using the non-informative priors in both the sub- and full-sample. Overall, under all distribution assumptions from Fig. 4 and Appendix F figures F1 and F2, those with the non-robust priors perform worse than the robust priors.

## The Fill-it-up-design in practice

In this section, we will illustrate how to proceed with the *Fill-it-up*-design and which practical and design implications are necessary. At first, we focus on the planning phase and a simulation study investigating the type I error probability in comparison to the MAP approach. Subsequently, we describe the final analysis to support researchers with its application. We consider the following scenario: Between September 15 in 2010, and November 21 in 2013, 605 patients were enrolled in the prospective international registry investigating the natural history of Friedreich’s ataxia [23]. The Scale for the Assessment and Rating of Ataxia (SARA) serves as a primary endpoint variable and was followed up yearly. In a fictive clinical trial with a two arm parallel group design, a new treatment should be compared to standard of care (SOC) with respect to the difference in mean SARA score two years after enrollment.

### Planning phase

Assume that $n_H=500$ patients from the registry received the definitive SOC, fulfill the same inclusion and exclusion criteria and showing at least a two year follow-up. In this scenario, one might think about using the registry data of the SOC-patients within the randomized clinical trial. For the application of the *Fill-it-up*-design we use the setting for equal type I error probabilities $\alpha _{{S_1}}=\alpha _{{S_2}}$ and balanced sample sizes $n_E=n_C$ and $n_E'=n_C'$ to establish an effect size $\delta =0.275$ at the overall power of 0.8. In Table 3 the design parameters required for the initial calculation of the required sample size for the *Fill-it-up*-design are summarized. Using equations (5) and (6) the *Fill-it-up*-design would be designed with $N_{FIU}=328$ and $\gamma N_{FIU}=192$ resulting in $\gamma =58.3\%$. In the further planning phase, the type I error probability for the equivalence pre-test is discussed i.e. $\alpha _{Ept} \in \{0.01, 0.05, 0.1, 0.2\}$ as well as the choice of expected values in the treatment groups.

**Table 3:** Choice of design parameters for the *Fill-it-up*-design

Parameter		Value
$n_H$	Sample size of historical control group *H*	500
$\delta$	Effect size	0.275
$1-\beta _{S_1}=1-\beta _{S_2}$	Power of ($S_1$) and ($S_2$)	0.8
$\alpha _{S_1}=\alpha _{S_2}$	Significance Level of ($S_1$) and ($S_2$)	0.05
$\alpha _{Ept}$	Significance Level of (*Ept*)	$\{0.01, 0.05, 0.1, 0.2\}$
$\Delta$	Equivalence Margin	$\left[ \sqrt{\frac{1}{n_H}+\frac{1}{n_C}} \cdot z_{1-\alpha _{Ept}} , \delta \right)$

### Simulation study

For the analysis of the type I error probability, a simulation study is carried out for which two different scenarios are considered. In Table 4 the choices of further design parameters used in the simulation study are summarized.

**Table 4:** Choice of design parameters for simulation study

Parameter		Scenario I	Scenario II
$\alpha _{Ept}$	Significance Level of Equivalence pre-test (*Ept*)	$\{0.01, 0.05, 0.1, 0.2\}$	
$\mu _C$	Expected response of control group *C*	0	0
$\mu _E$	Expected response of experimental group *E*	$-\omega \Delta$	0
$\mu _H$	Expected response of historical control group *H*	$- \Delta$	$3 \Delta$

The first scenario reflects the minimum case. This corresponds to the minimum type I error probabilities expected from the previous evaluations. This scenario thus describes the case in which the two control groups differ by exactly the selected margin and the treatment difference is $-\omega \Delta$. The second scenario reflects the maximum case. This corresponds to the maximum type I error probabilities expected from the previous evaluations. This scenario thus describes the case in which the two control groups differ substantially more than the selected margin and there is no treatment difference expected. For both scenarios, the equivalence margin was chosen according to (7) and therefore varies for the different choices of the significance level $\alpha _{Ept}$. We investigated the equivalence margin $\Delta =0.27$ for $\alpha _{Ept}=0.01$, $\Delta =0.22$ for $\alpha _{Ept}=0.05$, $\Delta =0.19$ for $\alpha _{Ept}=0.1$ and lastly $\Delta =0.15$ for $\alpha _{Ept}=0.2$. For all simulations settings 50 thousand simulation replications were modeled. Since we are concerned with a rather small treatment effect, as mentioned above, we can see that the number of people to be recruited in the first step is slightly higher than in the case of larger treatment effects. Average sample sizes are displayed in Table 5 for different scenarios of the significance levels $\alpha _{Ept}$. Compared to this the corresponding standard fixed sample design using the superiority test ($S_2$) would involve a sample size of 328 with a power of 0.8. All in all, this means that with the *Fill-it-up*-design we could save 136 patients in the newly planned study if we can show equivalence. This is also reflected in the average sample size, which depends mainly on the choice of $\alpha _{Ept}$. It can be said that the smaller $\alpha _{Ept}$ is chosen, the less the average sample size can be reduced with the help of the design. However, on the one hand, the relaxed significance level of 0.2 may be an interesting choice, but on the other hand, it is also the most restrictive setting concerning the equivalence margin. Regarding the type I error probabilities between the two designs, it can be stated that the MAP approach using the full-sample is superior to the family wise error rate of the *Fill-it-up*-design. Here, a smaller type I error probability is achieved in both scenarios. However, in this case, the maximum number of patients is needed to achieve these results, whereas the *Fill-it-up*-design can potentially save patients. However, one has to accept a small inflation of the error compared to the MAP approach. Looking at the type I error probability of the sub-sample in the MAP approach, we see that the *Fill-it-up*-design achieves smaller family wise error rates in Scenario I while the values approach each other as $\alpha _{Ept}$ increases. In all cases, however, the error probability is kept below 5$\%$. Finally, in Scenario II, we see slightly higher type I error inflations in the *Fill-it-up*-design, although the MAP approach can keep within the 5$\%$ bound in both the full- and the sub-sample.

**Table 5:** Simulation results of the MAP approach and Fill-it-up-design for an effect size of $\delta =0.275$ when $n_H=500$ and $N_{FIU}=328$, $\gamma N_{FIU} =192$ depending on the choice of the significance level of the equivalence pre-test $\alpha _{Ept}$ and corresponding choices of the equivalence margin $\Delta$

		FIU			MAP	
Scenario	$\Delta$	$\alpha _{Ept}$	AVN	FWER	TIE (full-sample)	TIE (sub-sample)
I	0.27	0.01	328	0.0002	0.0002	0.0013
	0.22	0.05	322	0.0015	0.0007	0.0029
	0.19	0.1	316	0.0033	0.0015	0.0043
	0.15	0.2	302	0.0072	0.0039	0.0074
II	0.27	0.01	328	0.0519	0.0461	0.0447
	0.22	0.05	322	0.0519	0.0454	0.0439
	0.19	0.1	316	0.0519	0.0453	0.0437
	0.15	0.2	302	0.0519	0.0447	0.0428

*AVN* Average sample size, *FWER* Family wise error rate, *TIE* Type I error

### Analysis

We now assume that the *Fill-it-up*-design is carried out as planned in Table 3. In the first step, 96 patients were randomized into the control group *C* and 96 patients in the experimental group *E*, resulting in $\gamma N_{FIU}=192$. Thus, a data set *fiudata* would be available in which the data of the 500 historical controls and the patients randomized in groups *C* and *E* in the first step are contained. For example, the data set can follow the form shown in Table 6. The subsequent R codes refer to a data set in this format.

**Table 6:** Exemplary format of the present data set *fiudata*

PatID	Group	Recruitment	Response
1	H	historical	...
...	...	...	...
500	H	historical	...
501	C	initial	...
...	...	...	...

A variable description of this data set is inserted in Table 7.

**Table 7:** Exemplary variable description of the present data set *fiudata*

Variable	Description	Characterisation
PatID	Patient ID in the database	Number
Group	Treatment group	H (historical), E (experimental), C (control)
recruitment	Recruitment status	initial (recruited in the first step), further (recruited in the second step), historical (recruited historically)
response	Response of primary endpoint	SARA Score

Listing 1 shows the parameters required for the following calculations. 
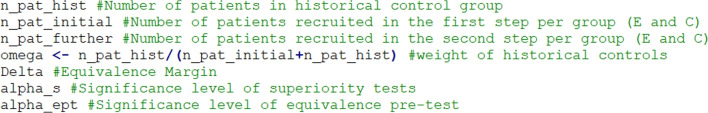


The equivalence test (*Ept*) is now performed by calculating the test statistic (1) and comparing it with the corresponding critical value. The test decision can be made in R using the R-code displayed in listing 2. 
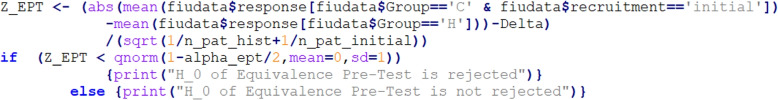


If the null hypothesis of the equivalence test is rejected, the two control groups are considered comparable and in the next step, the final superiority test (*S*1) is performed without further recruitment of patients. This is again done by calculating the test statistic from formula 2 and the associated critical value, as shown in listing 3.
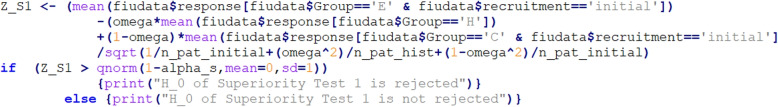


If the null hypothesis of the equivalence test is not rejected, the historical controls are discarded. In addition, 68 patients are now randomized to each of groups *E* and *C*, so that the previously calculated sample size of $N_{FIU}=328$ is achieved. The dataset *fiudata* is thus extended by these patients and finally superiority test (*S*2) is performed. This is again done by calculating the test statistic from formula 3 and the associated critical value, as shown in listing 4.
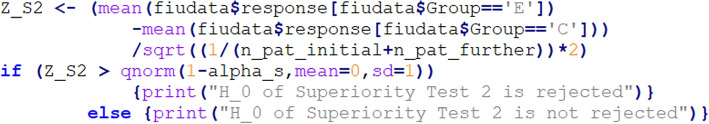


## Recommendations

As most researchers are sceptic about borrowing external information for a randomized clinical trial, some conditions might be reasonable before using the *Fill-it-up*-design. To avoid the use of external data biasing the estimate of the treatment effect, the data should be comparable to the new data, which must be ensured in the identification and selection process in addition to the equivalence pre-test. It is unavoidable to place restrictions on the historical data before the current study can be planned. Overall, this should be ensured through compliance with inclusion and exclusion criteria, verification of the quality of the data and the data source, and the planning process. With consideration of the discussion in Kopp-Schneider [16], we propose the following framework:

According to the underlying question to be answered by the analysis, all relevant patient and disease-specific variables that need to be collected for the historical control group *H* must be documented. Further important variables to be collected in the two randomized groups *E* and *C *have to be identified and also required from the historical database. Additionally to the required variable list, essential inclusion and exclusion criteria, again with regard to patient and disease-specific fields, need to be defined before screening the registry data. When these cornerstones have been set appropriate registries have to be screened to identify possibly available historical control data. For this step, we suggest that a similar process should be followed as specified for systematical reviews and meta-analyses in the PRISMA statement [18]. From adequate registry data which fulfills the inclusion and exclusion criteria from a suitable database, the random sample of historical controls is composed. In an ideal case, this dataset should remain blinded to all investigators until the equivalence pre-test is performed. Only the blinded study committee, which can for example be considered as a data safety committee, will take over the study planning to show the desired treatment effect based on the knowledge of the sample size $n_H$. In practice, it is important to get as close as possible to this best-case scenario to ensure the most optimal study planning. Both the required sample sizes of the first step of our design and the potential sample size of the second step are calculated to determine the desired treatment effect. All these steps should therefore be completed before recruitment and data collection of the new randomized part of the study. The specific inclusion and exclusion criteria must therefore be chosen in a way that ensures the potential comparability of historical and new data and of course the data quality. To this purpose, we would suggest that the six criteria of Pocock [21] are followed to ensure the safe use of historical controls:

1. The standard treatment has to be precisely defined and must be the same treatment for randomized controls.
2. The historical control group must have been part of a clinical study with the same requirements for patient eligibility.
3. The methods of treatment evaluation have to be the same.
4. Patient characteristics have to be comparable.
5. The study must have been performed in the same organization with the same investigators.
6. There should be no indications leading one to expect a difference.

These criteria are still to be considered as proper restrictions nowadays [8]. Although these might be very rigorous and restrictive, it makes sense to follow them and only deviate from the conditions by giving an adequate reason. To increase transparency, we also recommend that it is clearly stated in percentages how much of the total information comes from the randomized evidence and how much from the historical controls.

## Conclusion

### Discussion

Viele et al. [30] investigated in their “test-then-pool” procedure a different approach, i.e. to use the historical controls unless the pre-test on the difference of the expected response of the randomized and historical controls is not rejected, i.e. the test on $H_0: | \mu _C - \mu _H | = 0$ versus $H_1: | \mu _C - \mu _H | \not = 0$. Note that he recommended a type I error probability of $\alpha =0.1$ or $0.2$ for the pre-test. This test is rather an optimistic perspective as the test assumes the comparability of the two control groups as long as the comparability hypothesis is rejected. Without power control, this could not be associated with the equivalence test [27]. However, Viele et al. [30] stated that the superiority pre-test could be substituted by an equivalence test. But this approach has not yet been evaluated.

The equivalence pre-test incorporated in the *Fill-it-up*-design reflects a more sceptical perspective, that the comparability of the two control groups has to be proven. Moreover, the application of an equivalence test needs the specified formulation of an equivalence margin $\Delta$, which quantifies the degree of comparability. Contrary to the recommendation of Viele et al. [30] to perform the pre-test with a more relaxed significance level, we have found that this requires larger sample sizes to maintain the overall power at 0.8. We found that the family wise error rate is maximized for larger differences between the expected response of the historical and current controls and for a weighted treatment effect of zero while maintaining it at $5\%$ under the respective null hypotheses $H_0^{Ept}$ and $H_0^{S_1}$. Additionally, we conclude, that the choice of equivalence margin plays an essential role. As we have examined in our calculations, this must not be too small to be able to reject the null hypothesis. In contrast, however, it should of course not reach the level of the effect size.

Using historical controls only makes sense if we have some robust prior belief that it will be very close to the current controls. The pre-test cannot replace this assumption, it can just serve as a safety net if the assumption of equality of historical and current controls is completely wrong. We have also seen that the maximum sample size of the design is larger than that required for the one-step approach without the inclusion of historical controls. Further more the sample size increases as the heterogeneity between the two control groups, i.e. historical and concurrent controls, increases. This reflects the premium to be paid for using the design when equivalence cannot be achieved.

Compared to the existing MAP approach, we were able to show that the *Fill-it-up*-design can achieve comparable results in terms of evaluation of type I error probability and power. In some situations, a slight type I error inflation might be considered.

Overall, there is a growing interest in the inclusion of historical control data. The inclusion of non current controls allows, on the one hand, more studies to be carried out in small population groups if the sample size can be reduced. On the other hand, the use of this method can also be considered for more complex trials such as platform trials [17]. The *Fill-it-up*-design should, according to our investigations, be considered as a way out in exceptional situations where a hybrid design is deemed necessary.

### Limitations and generalizations

As a distinct limitation of the *Fill-it-up*-design, it should be noted that the combination of the hypotheses of the equivalence test (*Ept*) and the superiority test with included historical controls ($S_1$) leads to a weaker conclusion than the superiority test without historical controls ($S_2$) alone. This is due to the fact that the intersection hypothesis of the first case from (*Ept*) and ($S_1$) results strictly speaking in a non-inferiority conclusion between the groups *E* and *C* rather than in a superiority conclusion. With the *Fill-it-up*-design we are only able to investigate the superiority of the experimental group *E* versus the combined control group *C*+*H*. However, when historical data is included, there remains another side to the story.

The approach can be extended to unbalanced allocation ratios $n_E/n_C \not = 1$. It should be noted that a slight imbalance will be observed most often in practice. However, as with continuous endpoints, a slight imbalance will have an ignorable effect on the power of the trial, so that the balanced case evaluation gives direction for the practice. Further, as the known variance case is rather rare in practical situations, it might be sufficient for the investigation of large sample properties. In addition, the design requires further investigation concerning distributional assumptions and differences between the groups, such as the possibility of differences in variances. Initially, we focussed on the use of the z-test. However, the approach described in the paper can be directly followed to derive the properties, when using the corresponding *t*-distribution of the test statistics rather than the normal distributions. Moreover, the use of bootstrap techniques for resampling could be considered in order to estimate the respective statistics more robustly.

A generalization of the *Fill-it-up*-design would be to implement a sample size reassessment step based on the already observed randomized data, in the case the null hypothesis of the equivalence test could not be rejected, meaning that comparability could not be established. This potentially reduced the cost of the *Fill-it-up*-design even in the case of ignoring the historical controls. When allowing for sample size reassessment the incorporation of a futility stop should be taken into account as well. This has implications on the whole setting, in particular the derivation in the sections entitled “Type I error probability of the Fill-it-up-design” and “Power of the Fill-it-up-design”.

Furthermore, it should be mentioned that the comparability of the two control groups is of course difficult to establish based on the outcome variable only. Therefore, it is also necessary to consider the comparability of the possible baseline characteristics. On the one hand, this issue can be considered through the information provided in the “Recommendations” section. On the other hand, the statistical analysis of data obtained from trials using the *Fill-it-up*-design could also be extended to more complex models, to be able to adjust for confounders.

In this paper, the *Fill-it-up* design was evaluated as a test problem. In order to evaluate it in terms of estimation methods, additional operational characteristics such as bias and mean squared error need to be addressed.

Further possible extensions of the design are generalizations to other endpoints as binary and time to event measurements. In addition, one could include not only one set of historical controls but consider multiple historical data sets.

## Supplementary Information

Supplementary Material 1

## Data Availability

The datasets generated and analyzed during the current study available from the corresponding author on reasonable request.
