# Supplementary material for: Evaluation of the Fill-it-up-design to use historical control data in randomized clinical trials with two arm parallel group design
Source: BMC Med Res Methodol. 2024 Sep 9;24:197. doi: 10.1186/s12874-024-02306-2 (PMC11382532; doi:10.1186/s12874-024-02306-2)
Supplement: Supplementary file 1 — Supplementary Material 1 [file 12874_2024_2306_MOESM1_ESM.pdf]

Appendix A Optimization and Covariances

$$\frac{d}{d\omega}\left(\frac{\omega^2}{n_H}+\frac{(1-\omega)^2}{n_C}\right)=\frac{2\omega}{n_H}-\frac{2}{n_C}+\frac{2\omega}{n_C}=2\omega\left(\frac{1}{n_H}+\frac{1}{n_C}\right)-\frac{2}{n_C}=0$$
$$\rightarrow \omega^*=\frac{n_H}{n_H+n_C} \tag{A1}$$

$$\begin{aligned}Cov(Z^*_{S_1},Z_{Ept}) &= Cov\left(\frac{\bar{y}_E-(\omega^*\bar{y}_H+(1-\omega^*)\,\bar{y}_C)}{\sqrt{\frac{1}{n_E}+\frac{1}{n_C+n_H}}},\frac{|\bar{y}_C-\bar{y}_H|-\Delta}{\sqrt{\frac{1}{n_H}+\frac{1}{n_C}}}\right)\\ &= \frac{1}{\sqrt{\frac{1}{n_E}+\frac{1}{n_C+n_H}}\sqrt{\frac{1}{n_H}+\frac{1}{n_C}}}\\ &\qquad\cdot Cov\left(\bar{y}_E-(\omega^*\bar{y}_H+(1-\omega^*)\,\bar{y}_C)\,,|\bar{y}_C-\bar{y}_H|-\Delta\right)\\ &= \sqrt{\frac{n_En_Cn_H}{n_C+n_H+n_E}}\left(-\frac{\omega^*}{n_H}+\frac{1-\omega^*}{n_C}\right)=0.\end{aligned} \tag{A2}$$

$$\begin{aligned}Cov(Z_{S_2},Z_{Ept}) &= Cov\left(\frac{\bar{y}'_E-\bar{y}'_C}{\sqrt{\frac{1}{n_E+n'_E}+\frac{1}{n_C+n'_C}}},\frac{|\bar{y}_C-\bar{y}_H|-\Delta}{\sqrt{\frac{1}{n_H}+\frac{1}{n_C}}}\right)\\ &= \sqrt{\frac{(n_E+n'_E)(n_C+n'_C)n_Cn_H}{((n_C+n'_C)+(n_E+n'_E))(n_C+n_H)}}\left(Cov\left(\bar{y}_C,\bar{y}'_C\right)\right)\\ &= \sqrt{\frac{(n_E+n'_E)(n_C+n'_C)n_Cn_H}{((n_C+n'_C)+(n_E+n'_E))(n_C+n_H)}}\left(\frac{n_C}{(n_C+n'_C)n_C}\right)\\ &= \sqrt{\frac{(n_E+n'_E)n_Cn_H}{((n_C+n'_C)+(n_E+n'_E))(n_C+n'_C)(n_C+n_H)}}.\end{aligned} \tag{A3}$$

Appendix B Derivation of Family wise error rate

The first component of the family wise error rate (FWER) of the family of hypothesis $H_0^{Ept}, H_0^{S_1}, H_0^{S_2}$ can be calculated from

$$\begin{aligned}\alpha_{Ept,S_1} = &\int_{z_{1-\alpha_{S_1}}}^{\infty} \int_{-\infty}^{-z_{1-\frac{\alpha_{Ept}}{2}}} f_{(Z_{Ept},Z_{S_1})} dz_{Ept} dz_{S_1} \\ &+ \int_{z_{1-\alpha_{S_1}}}^{\infty} \int_{z_{1-\frac{\alpha_{Ept}}{2}}}^{\infty} f_{(Z_{Ept},Z_{S_1})} dz_{Ept} dz_{S_1}.\end{aligned} \tag{B1}$$

In the special case where $\omega = \omega^*$, since Z_{Ept} and $Z_{S_1}^*$ are uncorrelated, $f_{(Z_{Ept},Z_{S_1})}$ is obtained as the product of normal densities by

$$\begin{aligned}f_{(Z_{Ept},Z_{S_1})} := &\,\phi\left(\frac{z_{Ept} - \mu_{Ept}}{\sigma_{Ept}}\right) \cdot \phi\left(\frac{z_{S_1^*} - \mu_{S_1^*}}{\sigma_{S_1^*}}\right) \\ = &\,\frac{1}{\sqrt{2\pi}} \exp\left(-\frac{1}{2}\left(z_{Ept} - \frac{|\mu_C - \mu_H| - \Delta}{\sqrt{\frac{1}{n_C} + \frac{1}{n_H}}}\right)^2\right) \\ &\cdot \frac{1}{\sqrt{2\pi}} \exp\left(-\frac{1}{2}\left(z_{S_1} - \frac{\mu_E - (\omega\mu_H + (1-\omega)\mu_c)}{\sqrt{\frac{1}{n_E} + \frac{\omega^2}{n_H} + \frac{(1-\omega)^2}{n_C}}}\right)^2\right).\end{aligned}$$

The second component of the family wise error rate α_{Ept^c,S_2} is the joint rejection probability of the superiority test (S_2) and the non-rejected equivalence pre-test. This can be calculated from

$$\alpha_{Ept^c,S_2} = \int_{z_{1-\alpha_{S_2}}}^{\infty} \int_{-z_{1-\frac{\alpha_{Ept}}{2}}}^{z_{1-\frac{\alpha_{Ept}}{2}}} f_{(Z_{Ept},Z_{S_2})} dz_{Ept} dz_{S_2}, \tag{B2}$$

where $f_{(Z_{Ept},Z_{S_2})}$ is a bivariate normal density with mean

$$\boldsymbol{\mu_{Ept,S_2}} := \begin{bmatrix} \frac{|\mu_C - \mu_H| - \Delta}{\sqrt{\frac{1}{n_C} + \frac{1}{n_H}}} \\ \frac{\mu_E - \mu_C}{\sqrt{\frac{1}{n_E + n'_E} + \frac{1}{n_C + n'_C}}} \end{bmatrix}$$

and covariance

$$\boldsymbol{\Sigma_{Ept,S_2}} := \begin{bmatrix} 1 & \sqrt{\frac{(n_E + n'_E) n_C n_H}{((n_C + n'_C) + (n_E + n'_E))(n_C + n'_C)(n_C + n_H)}} \\ \sqrt{\frac{(n_E + n'_E) n_C n_H}{((n_C + n'_C) + (n_E + n'_E))(n_C + n'_C)(n_C + n_H)}} & 1 \end{bmatrix}$$

obtained by

$$f_{(Z_{Ept},Z_{S_2})} := \frac{\exp\left(-\frac{1}{2}\left(\begin{bmatrix} z_{Ept} \\ z_{S_2} \end{bmatrix} - \boldsymbol{\mu_{Ept,S_2}}\right)^T \boldsymbol{\Sigma_{Ept,S_2}}^{-1}\left(\begin{bmatrix} z_{Ept} \\ z_{S_2} \end{bmatrix} - \boldsymbol{\mu_{Ept,S_2}}\right)\right)}{\sqrt{(2\pi)^2 det(\boldsymbol{\Sigma_{Ept,S_2}})}}.$$

Appendix C Family wise error rate for small and large treatment effects

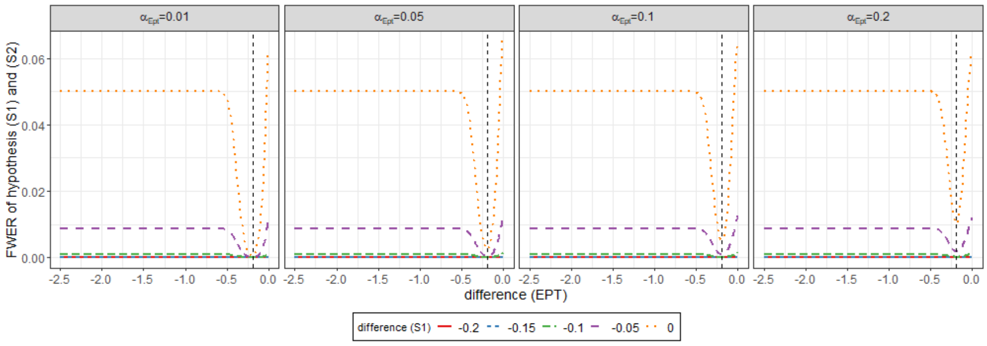

Fig. C1 Family wise error rate testing simultaneously superiority tests (S_1) and (S_2) for different scenarios of the Fill-it-up-design depending on the choice of the significance level of the equivalence pre-test. A small effect size $\delta = 0.2$ with $n_H = 500$ historical controls and an equivalence margin of $\Delta = 0.19$ is examined.

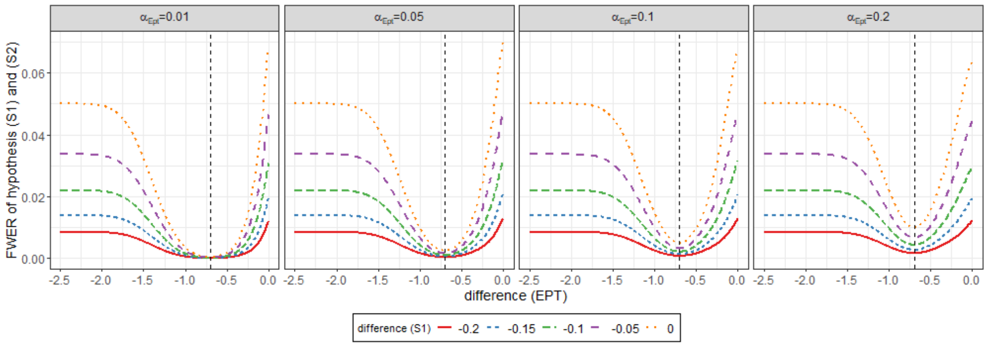

Fig. C2 Family wise error rate testing simultaneously superiority tests (S_1) and (S_2) for different scenarios of the Fill-it-up-design depending on the choice of the significance level of the equivalence pre-test. A large effect size $\delta = 0.8$ with $n_H = 500$ historical controls and an equivalence margin of $\Delta = 0.70$ is examined.

Appendix D Derivation of Power

β_{Ept,S_1} is the type II error probability for detecting equivalence in the pre-test without proving the difference in the superiority test ($S1$) and is obtained by

$$\beta_{Ept,S_1} = \int_{-\infty}^{z_{1-\alpha_{S_1}}} \int_{-\infty}^{-z_{1-\frac{\alpha_{Ept}}{2}}} f_{(Z_{Ept},Z_{S_1})} dz_{Ept} dz_{S_1} + \int_{-\infty}^{z_{1-\alpha_{S_1}}} \int_{z_{1-\frac{\alpha_{Ept}}{2}}}^{\infty} f_{(Z_{Ept},Z_{S_1})} dz_{Ept} dz_{S_1}. \tag{D1}$$

Respectively β_{Ept^c,S_2} is the type II error probability for not detecting equivalence in the equivalence pre-test and not proving the difference and is obtained by

$$\beta_{Ept^c,S_2} = \int_{-\infty}^{z_{1-\alpha_{S_2}}} \int_{-z_{1-\frac{\alpha_{Ept}}{2}}}^{z_{1-\frac{\alpha_{Ept}}{2}}} f_{(Z_{Ept},Z_{S_2})} dz_{Ept} dz_{S_2}. \tag{D2}$$

Appendix E Power and sample sizes for small and large treatment effects

Table E1 Overall power and sample sizes for different scenarios of the Fill-it-up-design depending on the choice of the equivalence margin Δ considering a small effect size $\delta = 0.2$ including $n_H = 500$ historical controls and significance levels $\alpha_{S_1} = \alpha_{S_2} = 0.05$.

α_{Ept}	Δ	N_{FIU}	γN_{FIU}	AVN	$1-\beta_{S_1}$ $= 1-\beta_{S_2}$	$1-\beta_{Ept,S_1}$	$1-\beta_{Ept^c,S_2}$	$1-\beta_{FIU}$
0.01	0.1946	620	400	618	0.80	0.9694	0.8310	0.8004
0.01	0.1973	620	400	618	0.80	0.9674	0.8327	0.8001
0.05	0.1376	620	400	610	0.80	0.9545	0.8488	0.8033
0.05	0.1680	638	414	628	0.81	0.9539	0.8464	0.8004
0.10	0.1049	656	426	634	0.82	0.9508	0.8511	0.8019
0.10	0.1485	796	538	772	0.88	0.9405	0.8598	0.8004
0.20	0.0696	638	414	594	0.81	0.9289	0.8747	0.8036
0.20	0.1340	676	442	630	0.83	0.8808	0.9193	0.8001

α_{Ept}: Two-sided significance level of equivalence pre-test, N_{FIU}: Maximum sample size of the Fill-it-up-design, γN_{FIU}: Sample size of the first stage of the Fill-it-up-design, β_{S_1}: Type II Error Probability superiority test ($S1$), β_{Ept,S_1}: Type II Error Probability of equivalence pre-test and superiority test ($S1$), β_{Ept^c,S_2}: Type II Error Probability of equivalence pre-test and superiority test ($S2$), $1-\beta_{FIU}$: Power of the Fill-it-up-design.

Table E2 Overall power and sample sizes for different scenarios of the Fill-it-up-design depending on the choice of the equivalence margin Δ considering a large effect size $\delta = 0.8$ including $n_H = 500$ historical controls and significance levels $\alpha_{S_1} = \alpha_{S_2} = 0.05$.

α_{Ept}	Δ	N_{FIU}	γN_{FIU}	AVN	$1-\beta_{S_1}$ $= 1-\beta_{S_2}$	$1-\beta_{Ept,S_1}$	$1-\beta_{Ept^c,S_2}$	$1-\beta_{FIU}$
0.01	0.6796	44	24	44	0.83	0.9973	0.8032	0.8005
0.01	0.7152	54	28	54	0.90	0.9902	0.8102	0.8004
0.05	0.4805	46	24	46	0.85	0.9628	0.8376	0.8004
0.05	0.6229	54	28	54	0.90	0.8566	0.9436	0.8002
0.10	0.3906	40	22	40	0.80	0.9979	0.8021	0.8000
0.10	0.5800	48	26	46	0.86	0.8576	0.9425	0.8002
0.20	0.2364	50	26	46	0.88	0.9202	0.8818	0.8020
0.20	0.5182	48	26	44	0.86	0.8368	0.9632	0.8000

α_{Ept}: Two-sided significance level of equivalence pre-test, N_{FIU}: Maximum sample size of the Fill-it-up-design, γN_{FIU}: Sample size of the first stage of the Fill-it-up-design, β_{S_1}: Type II Error Probability superiority test ($S1$), β_{Ept,S_1}: Type II Error Probability of equivalence pre-test and superiority test ($S1$), β_{Ept^c,S_2}: Type II Error Probability of equivalence pre-test and superiority test ($S2$), $1-\beta_{FIU}$: Power of the Fill-it-up-design.

Appendix F Evaluation of MAP approach

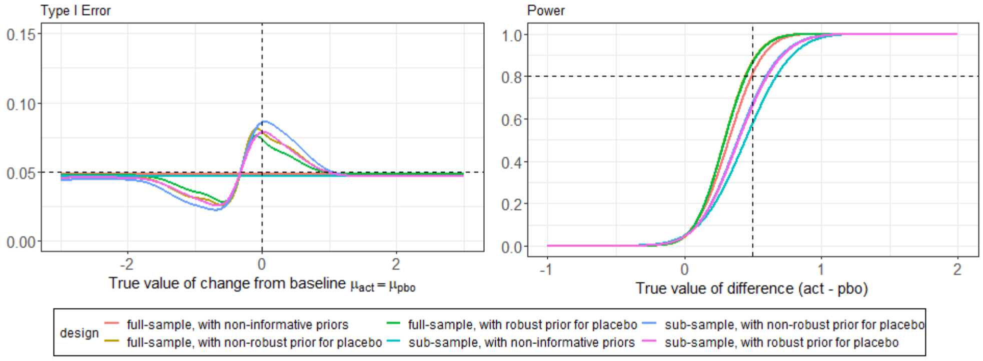

Fig. F1 Evaluation of Type I Error and Power for MAP approach for full-sample and sub-sample using non-informative, non-robust and robust priors and truncated normal distribution for heterogeneity.

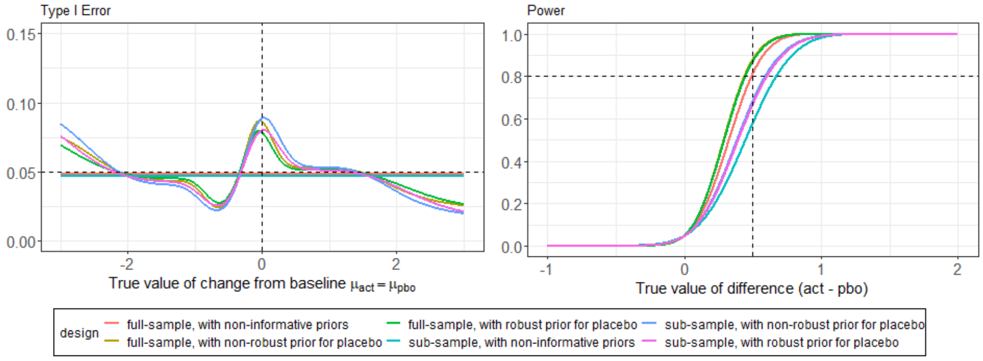

Fig. F2 Evaluation of Type I Error and Power for MAP approach for full-sample and sub-sample using non-informative, non-robust and robust priors and truncated cauchy distribution for heterogeneity.
